# Gadolinium contrast agents: dermal deposits and potential effects on epidermal small nerve fibers

**DOI:** 10.1007/s00415-023-11740-z

**Published:** 2023-05-04

**Authors:** Heidrun H. Krämer, Patrick Bücker, Astrid Jeibmann, Henning Richter, Angela Rosenbohm, Johanna Jeske, Panoraia Baka, Christian Geber, Matthias Wassenberg, Tanja Fangerau, Uwe Karst, Anne Schänzer, Christoph van Thriel

**Affiliations:** 1 Department of Neurology, Justus Liebig University of Giessen, 35392 Giessen, Germany; 2 Institute of Inorganic and Analytical Chemistry, University of Münster, 48149 Münster, Germany; 3 Institute of Neuropathology, University Hospital Münster, 48149 Münster, Germany; 4 Clinic for Diagnostic Imaging, Diagnostic Imaging Research Unit (DIRU),Department of Clinical Diagnostics and Services, Vetsuisse Faculty, University of Zurich, 8057 Zurich, Switzerland; 5 Department of Neurology, University of Ulm, 89081 Ulm, Germany; 6 Department of Neurology, University Medical Center, 55101 Mainz, Germany; 7 DRK Pain Center Mainz, 55131 Mainz, Germany; 8 Institute of Neuropathology, Justus Liebig University Giessen, 35392 Giessen, Germany; 9 IfADo-Leibniz Research Centre for Working Environment and Human Factors, 44139 Dortmund, Germany

**Keywords:** Small nerve fiber, Pain, Skin biopsy, Laser ablation-inductively coupled plasma-mass spectrometric imaging, Intraepidermal nerve fiber density

## Abstract

Small fiber neuropathy (SFN) affects unmyelinated and thinly myelinated nerve fibers causing neuropathic pain with distal distribution and autonomic symptoms. In idiopathic SFN (iSFN), 30% of the cases, the underlying aetiology remains unknown. Gadolinium (Gd)-based contrast agents (GBCA) are widely used in magnetic resonance imaging (MRI). However, side-effects including musculoskeletal disorders and burning skin sensations were reported. We investigated if dermal Gd deposits are more prevalent in iSFN patients exposed to GBCAs, and if dermal nerve fiber density and clinical parameters are likewise affected. 28 patients (19 females) with confirmed or no GBCA exposure were recruited in three German neuromuscular centers. ISFN was confirmed by clinical, neurophysiological, laboratory and genetic investigations. Six volunteers (two females) served as controls. Distal leg skin biopsies were obtained according to European recommendations. In these samples Gd was quantified by elemental bioimaging and intraepidermal nerve fibers (IENF) density via immunofluorescence analysis. Pain phenotyping was performed in all patients, quantitative sensory testing (QST) only in a subset (15 patients; 54%). All patients reported neuropathic pain, described as burning (*n* = 17), jabbing (*n* = 16) and hot (*n* = 11) and five QST scores were significantly altered. Compared to an equal distribution significantly more patients reported GBCA exposures (82%), while 18% confirmed no exposures. Compared to unexposed patients/controls significantly increased Gd deposits and lower *z*-scores of the IENF density were confirmed in exposed patients. QST scores and pain characteristics were not affected. This study suggests that GBCA exposure might alter IENF density in iSFN patients. Our results pave the road for further studies investigating the possible role of GBCA in small fiber damage, but more investigations and larger samples are needed to draw firm conclusions.

**Supplementary Information:**

The online version contains supplementary material available at 10.1007/s00415-023-11740-z.

## Introduction

Small fiber neuropathy (SFN) is defined as a damage of small unmyelinated C and thinly myelinated A δ nerve fibers causing neuropathic pain with distal distribution and autonomic symptoms [1]. The underlying aetiology of SFN includes metabolic, toxic, autoimmune or genetic disorders. However, the aetiology of approximately 30% patients remains unknown and is characterized as idiopathic SFN (iSFN) [2–5]. Small nerve fiber function cannot be evaluated with neurological routine tests, such as nerve conduction studies (NCS), which only detect impairment of the fast-conducting A-α (motor NCS) and A-β (sensory NCS) fibers. Special methods, such as quantitative sensory testing (QST) are helpful and needed to evaluate small fiber function [6]. Analyzing intraepidermal nerve fiber density (IENFD) in small skin biopsies is a recommended morphometric technique to enable the diagnosis of SFN [7, 8].

Our recently published animal study showed that even exposure to macrocyclic contrast agents can be associated with neuropathological finding like a small nerve fiber pathology in humans. Mice treated with macrocyclic gadolinium (Gd)-based contrast agents (mGBCAs) and linear GBCA (lGBCA) showed Gd deposition in the skin and a significant reduction of IENFD compared to controls. Additionally, terminal axonal swelling was observed in animals treated with linear GBCA [9]. Alkhunizi et al. [10] showed that Gd could be found in the spinal cord and peripheral nerves in rats repeatedly exposed to linear and macrocyclic GBCAs. However, only the treatment with the lGBCA (gadodiamide) was associated with pain hypersensitivity.

Gd, a heavy metal of the lanthanide group, has been used as a base for contrast agents in magnetic resonance imaging (MRI) for the last three decades. As free ion, Gd can inhibit calcium channels through competitive binding and thereby disturbing Ca^2+^ homeostasis and mitochondrial functions [11]. Moreover, Gd activates and sensitizes the vanilloid receptor TRPV1 an important pain receptor in humans [12]. To overcome such toxicity, chelated forms of Gd, classified as linear or macrocyclic (ionic or non-ionic), have been manufactured and used in humans. In general, macrocyclic GBCAs are thermodynamically and kinetically more stable than linear GBCAs [13, 14]. Although GBCAs were supposed to have a convincing risk profile two tremendous crises, namely the (1) GBCA-associated nephrogenic systemic fibrosis in patients with kidney insufficiency in 2006 [15] and in 2014 when (2) Gd deposits in human brains after application of GBCAs were described [16]. These side-effects have been predominantly reported in patients treated with linear GBCAs. Moreover, “symptoms associated with gadolinium exposure” (SAGE) such as fatigue, musculoskeletal disorders, burning skin sensations have been reported to be more prevalent for linear than macrocyclic GBCAs [17].

Although clinical and pathological consequences of Gd retention in the brain or SAGE in general were still unclear, in 2017 the European Medicines Agency (EMA) embraced a precautional position in patients safeguard and marketing authorization and linear GBCAs were suspended in the EU [18], with the exception using few linear GBCAs for special applications. However, investigations on this topic are still of broad interest, as linear GBCAs are further used outside the EU, and macrocyclic GBCAs continue to be applied worldwide.

In this study, we aim to investigate if skin Gd deposits are more prevalent in patients with iSFN who have been exposed to GBCAs, and if an effect on IENFD and clinical parameters could be observed.

## Materials and methods

### Subject and samples

This prospective observational study was carried out in three German neuromuscular centers (Giessen, Ulm and Mainz). Inclusion criteria were definite SFN according to the NEURODIAB criteria [19]. Patients were included in the study if idiopathic SFN was confirmed by clinical, neurophysiological, laboratory and genetic investigations. Patients were excluded if they had clinical signs of large fiber involvement, pathological nerve conduction (NC) studies or an underlying aetiology for SFN was present. Besides metabolic causes, infectious diseases, immune-mediated and paraneoplastic syndromes, genetic syndromes such as sodium channelopathies, Fabry Disease and TTR amyloidosis were ruled out [20]. During a standardized interview, the iSFN patients were asked for GBCA exposure, how often GBCAs were applicate, and the time point of the last exposure. If the patients were unsure, the radiologist responsible for the MR examinations was contacted and type, brand and volume of GBCA applied was noted. If the GBCA exposure remained unknown the patients were excluded.

The final sample consisted of twenty-eight patients fulfilling the NEURODIAB criteria of definite SFN. Of these participants, 23 iSFN patients (82%) reported exposures to GBCAs (iSFNe) and 5 (18%) declared that they have never been exposed to GBCAs (iSFNne). These frequencies significantly (Chi^2^ = 11.6, *p* < 0.001) deviate from the expected equal distribution (14 cases/exposure group). Quantitative sensory testing (QST) could be performed in 15 patients (54%). The distribution across the GBCA exposure groups is given in Table 1. The reasons for the MRI examinations were heterogeneous including imaging of brain, joints, and pelvis. The gender and age distribution of these three groups as well as the respective values of the controls are given in Table 1.

**Table 1:** Demographic data and descriptive statistics for age and exposure to GBCA for iSFN patients/controls and frequencies distribution of quantitative sensory testing (QST)

Groups	Gender	*n*	Age		Exposure to GBCA	
			Mean	SD	Yes	No
iSFN	Female	19	48.1	10.1	14	5
	Male	9	49.2	11.1	9	0
Controls	Female	2	25.0	1.4	0	2
	Male	4	23.5	0.6	0	4
Total		34	45.0	13.2	23 (82%^a^)	5 (18%)
QST^*b*^	Yes	15	49.9	10.1	12 (42.9%)	3 (10.7%)
	No	13	48.8	9.4	11 (39.3%)	2 (7.1%)

GBCA gadolinium-based contrast agent, iSFN idiopathic small fiber neuropathy

^a^Calculated for the 28 iSFN patients

^b^Quantitative sensory testing applied in iSFN patients

All patients received a skin biopsy at the distal leg according to recommendations [8]. Additional skin biopsies from six healthy subjects without history for GBCA exposure or neuropathic pain were included in the study.

### Ethics and approval

The study was approved by the central Ethics Committee of the Justus-Liebig-University of Giessen (ethics approval number AZ 27/20) as well as the local ethic committees from the participating centers. The Ethics Committees approved the conducted experiments on human participants. Informed and written consent was obtained from all participants. The study was conducted according to the current version of the Guidelines for Good Clinical Practice and Helsinki Declaration of the World Medical Association.

### Quantifying intraepidermal nerve density (IENFD)

To determine the intraepidermal nerve fiber density (IENFD) standard procedures were performed. From each biopsy, sections were stained with antibody against Protein Gene Product (PGP) 9.5 a neuron-specific protein that labels axons in the peripheral nervous system [21, 22]. IENFD was determined according to published counting recommendations. For all analyses, IENFD were *z*-transformed ${(z}_{\mathrm{individual}}=\frac{{\mathrm{IENFD}}_{\mathrm{individual}}-{\mathrm{IENFD}}_{\mathrm{reference}}}{{\mathrm{SD}}_{\mathrm{reference}}} )$ using the age-and sex-matched reference values. IENFD was considered significantly “reduced” when it was below the 5% percentile of the reference data (zIENFD < 1.64) [7]. The investigators were blinded to the samples during the morphometric analysis.

### Elemental bioimaging of gadolinium deposits in skin samples

**Fig. 1:**
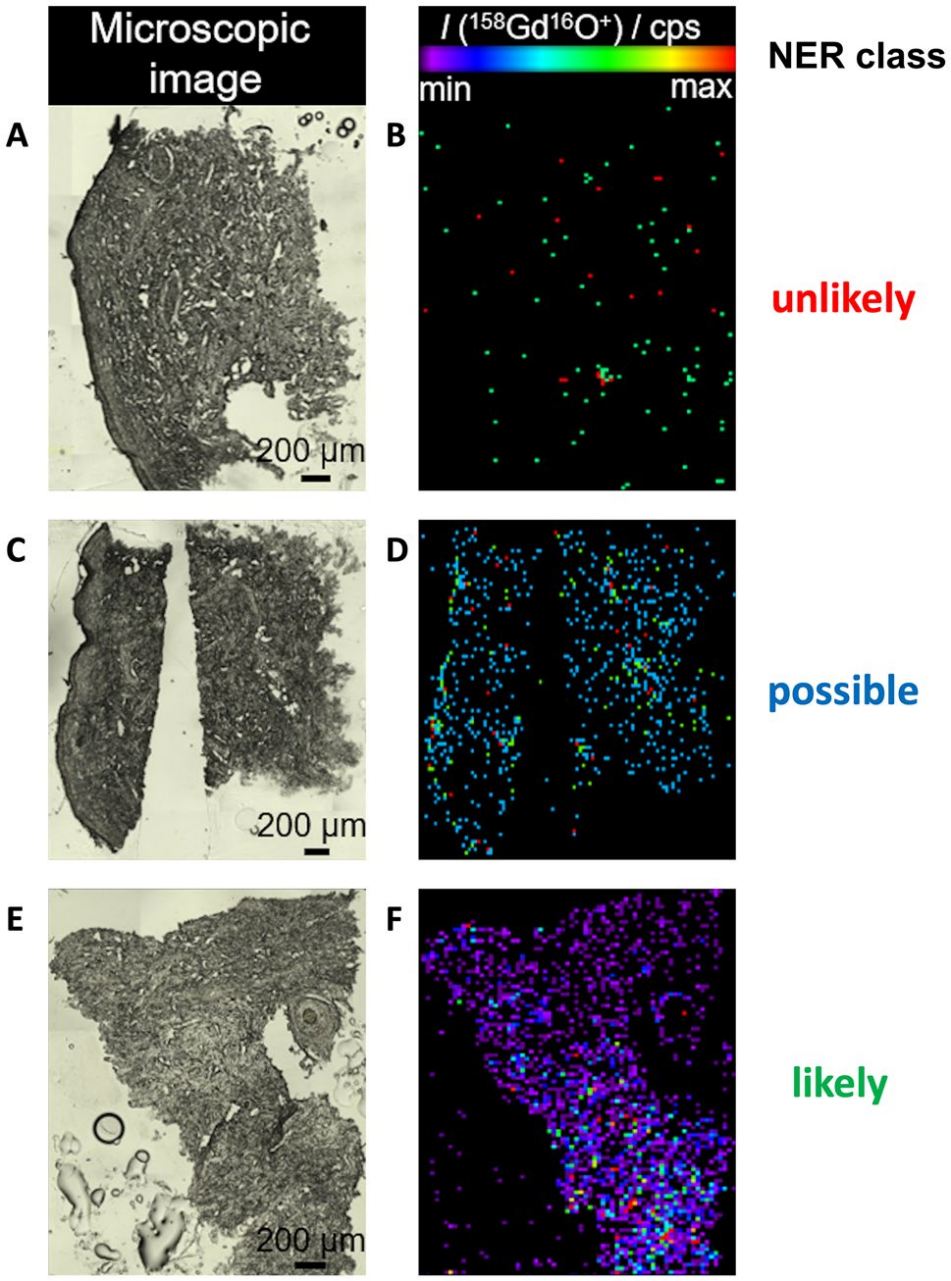
Microscopic images (**A**, **C**, **E**) of skin biopsy samples and the LA-ICP-TQMS- based detection of Gd (**B**, **D**, **F**) with a likelihood of prior GBCA injection being unlikely (**A**, **B**), possible (**C**, **D**), and likely (**E**, **F**). NER normalized event rate

From each skin biopsy sample, 10 µm thick cryosections were prepared and subjected to an elemental bioimaging procedure that can detect Gd in different organs [23] and that has been used in a previous animal study [9]. Skin Gd concentration was determined using laser ablation-inductively coupled plasma-mass spectrometric imaging (LA-ICP-MSI) as shown in Fig. 1. Laser ablation allows a subsequent spatially resolved elemental analysis via inductively coupled plasma-triple quadrupole mass spectrometry (ICP-TQMS) especially for metals in various tissues [23, 24]. A laser spot size of 25 µm and a corresponding stage speed of 100 µm/s were selected for high-throughput ablation. The formed aerosol is atomized in the plasma, and analyzed in the mass spectrometer, partly after reaction to the detected species (e. g., ^158^Gd^16^O^+^) in the triple quadrupole mass analyzer. Using an appropriate software package, the transient signal of the ICP-MS is used to reconstruct the spatial distribution of the analytes within the biopsy samples (Fig. 1).

To evaluate samples with overall low expected Gd concentrations, as in the case of human skin biopsies, a script-based semi-quantitative approach was developed, which introduces the Normalized Event Rate (NER) as an indicator for the real Gd concentration. Utilizing this value, all patients were classified, reflecting their likelihood of prior GBCA injection: lower than 3xstandard deviation (SD) of the controls (unlikely), greater than 3xSD and lower than 10xSD of the controls (possible), and greater than 10xSD of the controls (likely). For further information regarding the calculation of the NER, please refer to the supplemental material (Supplemental Material 1). To analyze possible group differences or associations, we used the frequency of patients within the classes (unlikely, possibly, and likely) as well as the individual NER as a more quantitative estimate of Gd in the tissue.

### Phenotyping of pain

#### Quantitative sensory testing (QST)

Quantitative sensory testing (QST) was performed according to the protocol of the German Research Network on Neuropathic Pain (DFNS) in 18 (55%) patients with iSFN [25, 26]. ISFN patients with or without Gd were compared to the normative data set of the German network on neuropathic pain (DFNS) and with each other [27]. A total of 11 parameters were used in the analyses: the thermal detection thresholds for the perception of cool (CDT) and warm (WDT), the thermal pain thresholds (cold pain threshold [CPT]; heat pain threshold [HPT]), the mechanical detection thresholds (MDT), the mechanical pain thresholds (MPT), a stimulus–response function for mechanical pain sensitivity (MPS), pain in response to light touch (dynamic mechanical allodynia [DMA]), the vibration detection threshold (VDT), the wind-up ratio (WUR) to assess pain summation to repetitive pinprick stimuli and the pressure pain threshold (PPT) at the thenar eminence.

QST data were *z*-transformed into a standard normal distribution (zero mean, unit variance) for each single parameter to allow a comparison of QST parameters independent of their physical units using the following expression (except DMA): *Z* = (value patient – mean controls)/SDcontrols. *Z*-scores below zero indicate a loss of function; *z*-scores above zero indicate a gain of function. Thus, elevations of thresholds (CDT, WDT, HPT, CPT, PPT, MPT, MDT, and VDT) result in negative z-scores, whereas increased ratings (MPS and WUR) result in positive z-scores.

#### Pain questionnaires

The current, maximum, and mean pain intensity of the last 4 weeks was obtained on a numeric rating scale in every SFN patients (anchors: 0 = no pain; 10 = worst pain imaginable). Pain quality and distribution was assessed using the German Pain Questionnaire of the German pain society as a section of the international society for the study of pain [28].

### Statistical analysis

For QST parameters, comparisons to the normative data were performed using t-tests as recommended [27]. Since only 2 patients reported DMA, no further analysis was calculated for this parameter. However, due to the small sample size bootstrapping (number of samples = 1000) procedure was used for the one sample t test using SPSS version 28.0 for Mac OS X (IBM Corp. Released 2021. IBM SPSS Statistics for Macintosh, Version 28.0. Armonk, NY, USA: IBM Corp). The iSFN patient with (iSFNe; exposed) and without (iSFNne; not exposed) confirmed GBCA exposure were compared by non-parametric Mann–Whitney-*U*-tests. Statistical evaluation of the LA-ICP-MSI-derived NERs and the *z*-transformed IENFD values was performed by non-parametric test (Kruskal–Wallis test followed by Dunn’s multiple comparisons test) using GraphPad Prism version 9.4.0 for Mac OS X (GraphPad Software, San Diego, CA, USA). Nominal or ordinal variables were analyzed by frequency tables and Chi^2^ tests as well as rank correlation analyses using SPSS version 28.0. For the analyses, the significance criterion was set to *p* = 0.05 and multiple comparisons were adjusted to the number of comparisons (Bonferroni correction).

### Data availability

Data are available in the tables.

## Results

### Patients’ GBCA exposure and pain characteristics

All patients had length-dependent clinical signs and symptoms of small nerve fiber damage and normal sural nerve conduction studies. 23 patients showed significantly reduced IENFD (*z* ≤ 1.64). From the five patients with normal IENFD, all patients presented with pathological thermal detection thresholds (*z* ≤ 1.96). Therefore, definite iSFN was diagnosed in all included 28 patients according to the NEURODIAB criteria [19].

The results of the standardized interviews about type, brand, duration since the last application, and injected volume of GBCA resulted in detailed data for 15 (65%) of the 23 iSNFe patients. This information together with the individual results of the elemental bioimaging and the z-scores of the intraepidermal nerve fiber density (IENFD) are given in Table 2.

**Table 2:** Detailed description of 15 iSFN patients with exposure to GBCA

P-Nr	Age	Type of GBCA	#Administrations	Dosage (ml)	Duration^a^	Compound	NER^b^	IENFD^c^
2	42	Linear	2	8, 15	7	Gadopentate, Gadopentate	27	− 2.015
14	53	Linear	1	18	10	Gadodiamide	8.9	− 1.663
19	44	Linear	1	12	13	Gadopentate	5.5	− 3.007
4	44	Macrocyclic	1	8	0.3	Gadobutrol	6.7	− 3.368
6	35	Macrocyclic	2	7. 15	0.6	Gadobutrol, Gadoterate	10	− 3.058
8	49	Macrocyclic	2	6, 14	1.5	Gadoterate, Gadobutrol	7.4	− 2.707
11	34	Macrocyclic	2	15, 15	0.33	Gadoterate, Gadoterate	4.8	− 2.328
25	46	Macrocyclic	2	12, 15	1	Gadobutrol, Gadoterate	28	− 2.990
28	61	Macrocyclic	2	7.5, 20	2.5	Gadobutrol, Gadoterate	6.0	− 2.496
29	47	Macrocyclic	1	15	3	Gadoterate	5.2	− 3.028
41	33	Macrocyclic	2	6, 12	3	Gadoterate, Gadobutrol	5.3	− 2.197
42	57	Macrocyclic	2	9, 10	4	Gadobutrol, Gadobutrol	53	− 3.211
24	59	Mixed	2	15, 20	4	Gadoterate, Gadodiamide	39	− 2.135
27	52	Mixed	4	12, 15, 15, 20	0.5	Gadoterate, Gadobenate, Gadoteridol, Gadoteridol	12	− 1.955
31	53	Mixed	2	7.5, 15	0.6	Gadopentate, Gadobutrol	20	− 1.777

^a^Duration (in years) from last GBCA treatment to biopsy

^b^Normalized event rate obtained from the LA-ICP-TQMS analyses (< 4.7: unlikely, > 4.7 and < 15.7: possible, > 15.7: likely)

^c^Age and gender-adjusted z-score of the IENFD

All patients presented length dependent neuropathic symptoms that are the clinical hallmarks of SFN. The mean symptom duration before diagnosis was 5.1 ± 4.5 years. Most of the patients described the sensation of pain as burning (*n* = 17), jabbing (*n* = 16) and hot (*n* = 11). All patients reported about neuropathic pain (pain intensity: current: 5.1 ± 3.2; mean within the last 4 weeks: 5.4 ± 2.7; maximum within the last 4 weeks: 7.2 ± 2.9). 14 of the iSFN patients were on pain medication at the time of biopsy. Their pain medication included Amitriptyline (*n* = 5), Duloxetine (*n* = 4), Gabapentine (*n* = 4), Lamotrigine (*n* = 3) and Cannabinoil oil (*n* = 2). The mean pain intensity did not differ between the two patient groups (mean pain intensity: iSFNe: 5.0 ± 2.8 vs. iSFNne: 6.8 ± 0.8; Mann–Whitney-*U*-test: 32.0, *p* = 0.14).

**Fig. 2:**
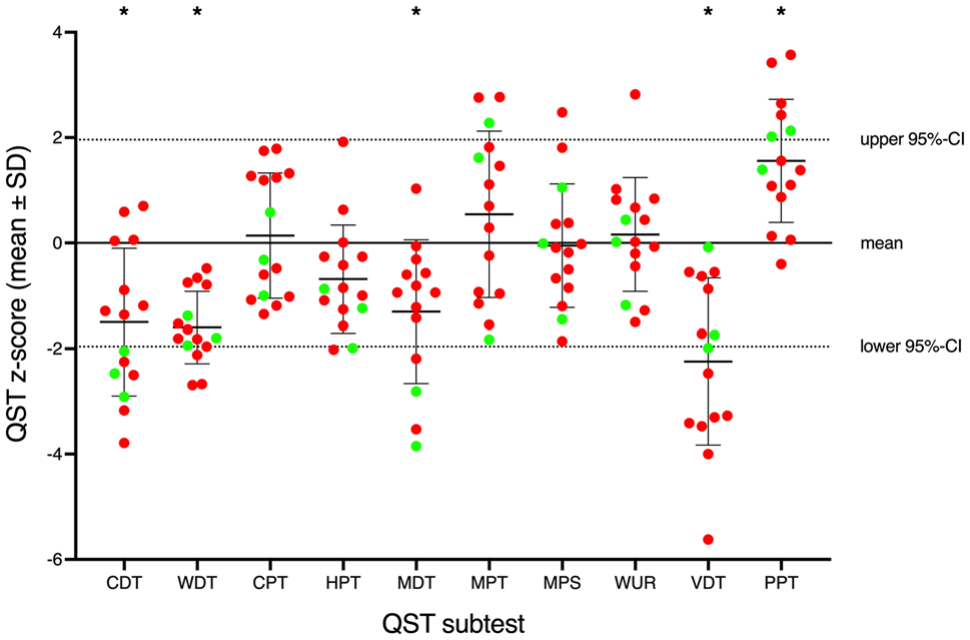
Individual, mean and standard deviation of the z-scores for the QST subtests obtained from 15 iSFN patients. Red dots indicate patients with confirmed GBCA application (iSFNe), while green dots represent patients without GBCA exposure (iSFNne). According to Magerl et al. (2010) *t* test were calculated to test significant differences to a matched control group created as a fictitious subpopulation of reference group and stars above the subtest labels indicates a Bonferroni corrected *p* value lower than 0.0045

QST examination that were available for 15 of our iSFN patients (54%) confirmed the findings of other SFN studies [29] (see Fig. 2).

The iSFN patients showed lower *z*-scores of the following subtest: CDT, WDT, VDT and PPT. The PPT *z*-score was significantly higher when [27] compared to normative data. Comparing QST data between iSFNe and iSFNne (red vs. green) showed no significant difference. One iSFNe and one iSFNne patient reported DMA.

### Patients’ elemental bioimaging of gadolinium deposits

All GBCA-related analyses were restricted to the 23 iSFNe, the 5 iSFNne patients and the 6 unexposed controls. The individual results of the elemental bioimaging analysis for all 28 iSFN patients and the 6 controls enrolled in this study can be found in Table 3.f female, m male^a^Yes = confirmed exposure, no = no exposure, c = controls^b^Compared to reference values Lauria et al.: reduced below 0.05 quantile values^c^Gd exposure according to the classifier (see Fig. 1)

**Table 3:** Morphometric and elemental bioimaging results of skin biopsies from patients with iSFN and healthy subjects

No.	P-Nr	Gender	Age at biopsy	Contrast agent exposure^a^	IENFD fibers/mm	Normal value 5% (median)^c^	Level of reduction^b^	*z*-Score	Gadolinium deposits (normalized event rate)	Gadolinium deposits (quantitative)^c^
1	1	f	44	Yes	2.8	5.7 (11.2)	Reduced	− 2.53	19.00	Likely
2	2	f	42	Yes	4.5	5.7 (11.2)	Reduced	− 2.02	27.00	Likely
3	4	f	44	Yes	0	5.7 (11.2)	Reduced	− 3.37	6.70	Possible
4	6	f	35	Yes	2.6	7.1 (12.4)	Reduced	− 3.06	10.00	Possible
5	8	f	49	Yes	2.2	5.7 (11.2)	Reduced	− 2.71	7.40	Possible
6	9	f	60	Yes	1.2	3.2 (8.7)	Reduced	− 2.26	7.10	Possible
7	11	m	34	Yes	0.24	5.2 (10.3)	Reduced	− 2.33	4.80	Possible
8	12	m	39	Yes	3.3	5.2 (10.3)	Reduced	− 2.00	5.50	Possible
9	14	f	53	Yes	4.27	4.3 (9.8)	Reduced	− 1.66	8.90	Possible
10	16	f	65	Yes	8.3	3.2 (8.7)	Normal	− 0.12	27.00	Likely
11	19	f	44	Yes	1.2	5.7 (11.2)	Reduced	− 3.01	5.50	Possible
12	24	m	59	Yes	1.2	3.5 (8.9)	Reduced	− 2.14	39.00	Likely
13	25	m	46	Yes	0.2	4.4 (9.6)	Reduced	− 2.99	28.00	Likely
14	27	f	52	Yes	3.3	4.3 (9.8)	Reduced	− 1.96	12.00	Possible
15	28	m	61	Yes	0	2.8 (8.3)	Reduced	− 2.50	6.00	Possible
16	29	f	47	Yes	1.13	5.7 (11.2)	Reduced	− 3.03	5.20	Possible
17	30	m	34	Yes	0.75	5.2 (10.3)	Reduced	− 3.10	9.90	Possible
18	31	m	53	Yes	3.1	3.5 (8.9)	Reduced	− 1.78	20.00	Likely
19	37	m	59	Yes	0.5	3.5 (8.9)	Reduced	− 2.80	30.00	Likely
20	41	f	33	Yes	5.36	7.1 (12.4)	Reduced	− 2.20	5.30	Possible
21	42	m	57	Yes	2.3	3.5 (8.9)	Reduced	− 3.21	53.00	Likely
22	44	f	41	Yes	2.4	5.7 (11.2)	Reduced	− 2.65	23.00	Likely
23	46	f	66	Yes	4.86	3.2 (8.7)	Normal	− 1.16	7.40	Possible
24	15	f	51	No	5.7	4.3 (9.8)	Normal	− 1.23	4.40	Unlikely
25	17	f	47	No	6.55	5.7 (11.2)	Normal	− 1.40	3.20	Unlikely
26	21	f	55	No	6.7	4.3 (9.8)	Normal	− 0.93	8.00	Possible
27	34	f	58	No	0.6	4.3 (9.8)	Reduced	− 2.77	9.40	Possible
28	36	f	57	No	2.27	4.3 (9.8)	Reduced	− 2.26	12.00	Possible
C1	101	f	24	No	10.16	8.4 (13.5)	Normal	− 1.08	3.90	Unlikely
C2	102	m	23	No	7.4	6.1 (10.9)	Normal	− 1.21	3.50	Unlikely
C3	103	f	26	No	9.22	8.4 (13.5)	Normal	− 1.39	3.50	Unlikely
C4	104	m	23	No	8.26	6.1 (10.9)	Normal	− 0.91	1.70	Unlikely
C5	105	m	24	No	8.16	6.1 (10.9)	Normal	− 0.94	1.60	Unlikely
C6	106	m	24	No	11.0	6.1 (10.9)	Normal	0.03	1.80	Unlikely

f = female, m = male; b: yes = confirmed exposure, no = no exposure, c = controls; c: Compared to reference values Lauria et al.: reduced below 0.05 quantile values; d: Gd exposure according to the classifier (see Figure 1).

**Fig. 3:**
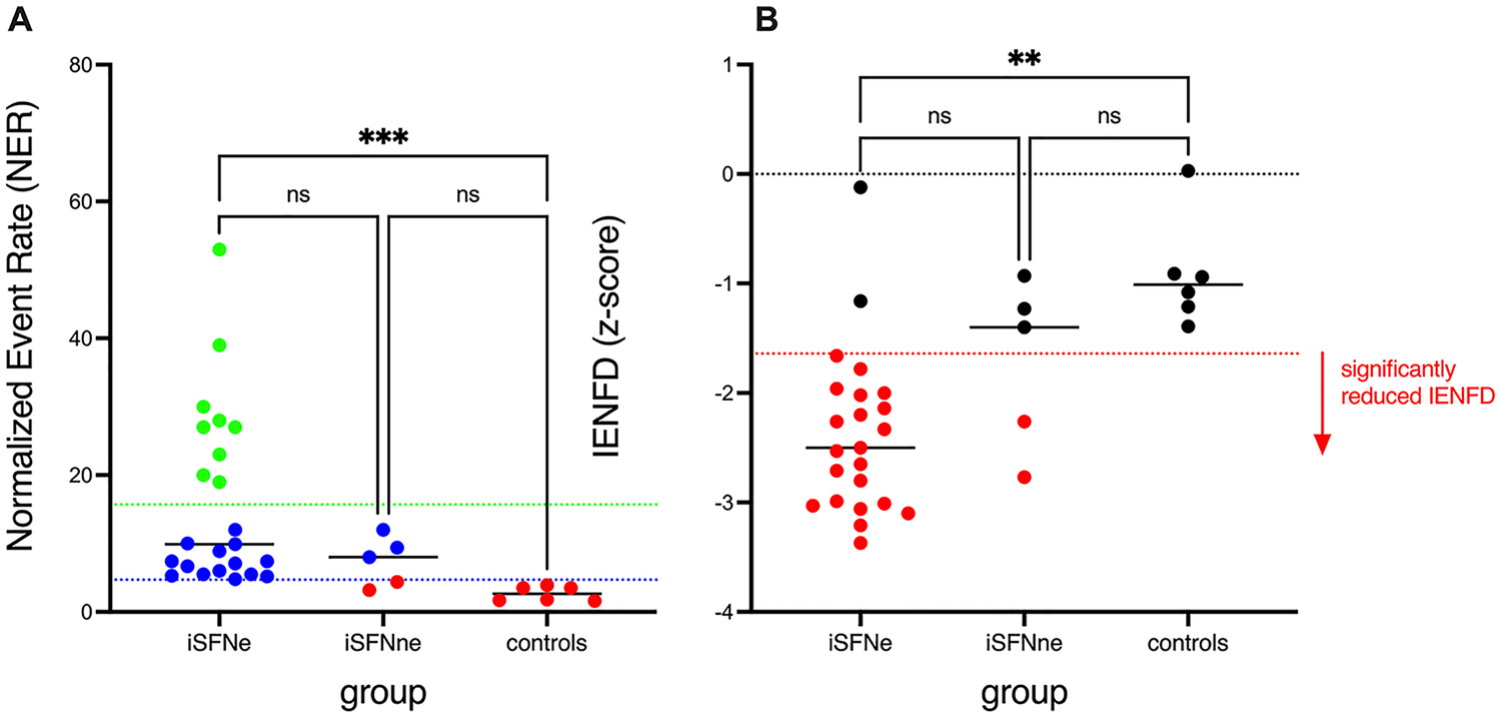
Chemical and neuropathological analyses of the skin biopsy samples of the iSFN groups (iSFNe: exposed to GBCA; iSFNne: not exposed to GBCA) and controls showing **A** the individual normalized event rates of Gd deposits obtained by LA-ICP-TQMS analysis, their medians (black lines) as well as the classifier results (red: unlikely; blue: possible; green: likely) and **B** individual and median (black lines) *z*-scores of intraepidermal nerve density (IENFD). *Z*-scores lower − 1.64 indicate a significant reduction (red dots in panel B) compared to normative data. Groups were compared by Dunn’s multiple comparisons tests (*p < 0.05, **p < 0.01, ***p < 0.001)

The application of the classifier approach (color-coded grouping in Fig. 3A) revealed that iSFNe patients were labelled as possible or likely, while in the iSFNne and controls were mainly classified as unlikely. Statistically, the three groups (*x*-axis in Fig. 3) differed significantly (Chi^2^: 24.06; *P* < 0.001) with respect to the results of NER-based classification (e.g. only possible or likely cases in the iSFNe group). This association was also confirmed by the ordinal-by-ordinal correlation resulting in Kendall’s tau-b of 0.54 (*P* < 0.001) indicating that a likely classification of the NER obtained in the elemental bioimaging analyses was associate with being in the iSFNe group.

**Fig. 4:**
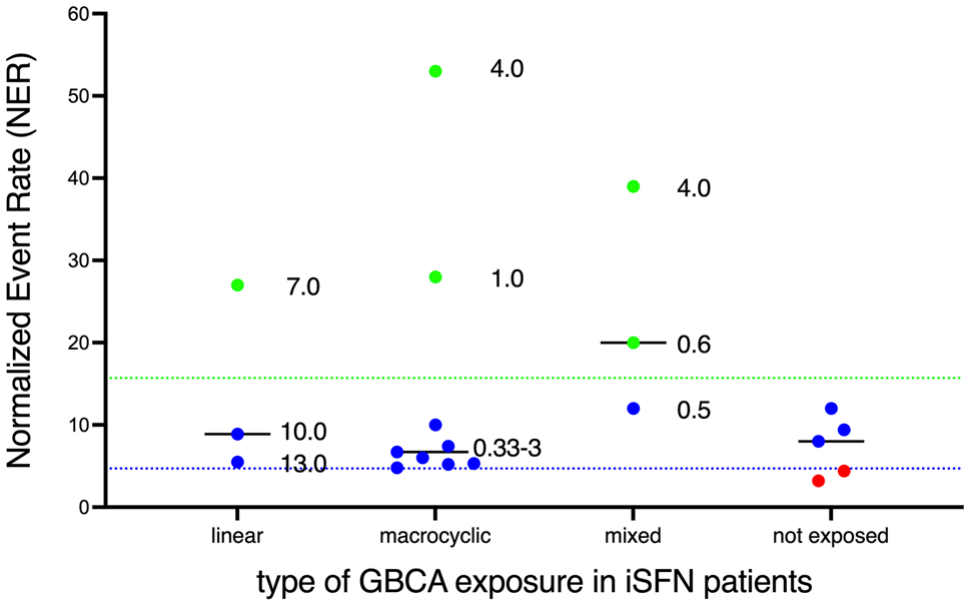
Individual measures and median values (black lines) of the normalized event rates derived from the LA-ICP-TQMS Gd detection in the skin biopsies from iSFN patients with exposures to linear or macrocyclic GBCAs only, mixed GBCAs exposures and those who were never exposed to any type of GBCA. The numbers close to the dots indicate the individual or range of the duration (in years) from the last GBCA injection to the skin biopsy. The classifier results (red: unlikely; blue: possible; green: likely) were used to label the individual NERs

Figure 3A also shows the quantitative results of the elemental bioimaging analyses of the Gd signals in the skin biopsy samples. Here, the mean rank values of the normalized event rates (NER) of the three groups were significantly different (Kruskal–Wallis statistic: 15.0; *p* < 0.001). Dunn’s multiple comparison tests yielded significant higher Gd deposits in skin samples of the iSFNe (GBCA exposed) patients compared to controls but no significant difference to the iSFNne (GBCA not exposed) patients could be statistically confirmed. iSFNne patients and healthy controls did not differ significantly with respect to their NERs. However, due to small number of patients with more detailed information about the type, dose, or duration since the last GBCA treatment (see Table 2) an in-depth analysis of this association was not possible (Fig. 4). However, neither the type of GBCA (linear vs. macrocyclic), nor the duration since the last application of the GBCA seems to be associated with the NER obtained by the elemental bioimaging approach.

### Patients’ intraepidermal nerve density (IENFD)

The z-transformed IENFD values of the three groups included in the GBCA-related analyses (iSFNe, iSFNne, controls) are shown in Fig. 3B. In Fig. 3B, the significant reduction is given as red dot (*z*-score < − 1.64) compared to black dots with normal IENFD values (*z*-score > − 1.64). In all patients with iSFN (iSFNe and iSFNne) 85% had a significantly reduced IENFD when compared to the reference data. In the control subjects and most of the iSFNne patients, the IENFD *z*-scores was in the normal range of the reference data [1, 7] The non-parametric analysis revealed a significant difference among the three groups (Kruskal–Wallis statistic: 12.9; *p* < 0.001) and Dunn’s multiple comparisons showed that the IENFD *z*-scores of the iSFNe patients were significantly lower than in controls. Even though a huge difference between the iSFNe and iSFNne for the IENFD *z*-scores is shown in Fig. 3B Dunn’s multiple comparisons could not confirm significance between the two groups.

The analysis of the binary IENFD scores (significantly vs. not significantly reduced; red vs. black dots in Fig. 3B) revealed that in iSFNe patients (*n* = 23) 21 patients (91.3%) showed significantly reduced IENFD *z*-scores while in iSFNne patients (*n* = 5) only two patients (40.0%) had a IENFD *z*-scores below − 1.64. Accordingly, the respective odds ratio quantifying the strength of the association between GBCA exposure and significant IENFD reduction is 15.8 (95% CI 1.6–157.6; *P* = 0.01) indicating an almost 16-fold higher risk for reduced IENFD *z*-scores in iSFN patients that previously received a GBCA during MRI examination.

Testing the association of the z-transformed IENFD with the QST subtests by rank correlations (Spearman’s rho) showed only one significant correlation of rho = − 0.57 (*p* = 0.03) between the IENFD z-score and the mechanical pain thresholds (MPT). However, when adjusting for multiple comparisons this association is no longer significant.

## Discussion

Gadolinium has been used for contrast agents in magnetic resonance imaging (MRI) for decades. In recent studies Gd deposits have been detected in several organs, including the brain [14, 15]. In animal studies, a neurotoxic effect of Gd on small nerve fibers has been reported [10] but human studies are lacking. In our study, we analyzed dermal Gd deposits from patients with confirmed iSFN and healthy volunteers with LA-ICP-MSI, which allows a spatially resolved element analysis for metals in various tissues [23, 24]. With this method, small amounts of Gd can be detected [24]. We were able to detect higher dermal Gd deposits in iSFN patients with confirmed GBCA exposure compared to healthy controls without exposure to GBCA. A modulating effect of Gd deposits on small nerve fibers in patients with iSFN can be assumed. The iSFN patients and controls were not matched with respect to age and for the IENFD an impact of this difference could be avoided using age-adjusted *z*-scores. As a rare earth element environmental exposure to gadolinium is unlikely and therefore, the results of the normalized event rates of Gd deposits obtained by LA-ICP-TQMS analysis might not be affected by the age differences. The non-significant difference between the iSFNne and controls in the NER might serve a support. However, further studies need larger sample to confirm this age independence of Gd deposits in the skin of humans.

In our study, we included only patients with iSFN that was diagnosed after a thorough work-up. The aim of the study was to analyze the presence of dermal Gd deposits in patients with iSFN and therefore we included patients with the diagnosis of definite iSFN. The analysis of IENFD is the gold standard for the diagnosis of SFN. However, QST analysis is still justified and pathological results allow the diagnosis of definite SFN even if the IENFD is still within normal range [19]. It is known from previous studies that IENFD further decreases over time resulting in a reduced IEFND at a later stage of the disease [30]. The recruitment of only definite SFN together with our rigid diagnostic regimen to identify truly idiopathic SFN to minimize the risk of other diseases causing the reported length-dependent symptoms of small fiber damage are the prerequisites to discuss a possible impact of Gd deposits on small fiber function. Therefore, our choice of included patients allows valid conclusions.

Other underlying aetiology for SFN were thoroughly ruled out [5]. Clinical criteria as neuropathic pain was confirmed in all patients with pain questionnaire. QST was performed in a subset of patients which showed in pathological results in all of them.

This procedure allows to argue for a possible role of Gd or GBCA deposits in small fiber damage in our iSFN patients, as suggested in animal studies [10]. Accordingly, in skin biopsies with more likely dermal Gd deposits the IENFD was significantly lower compared to patients with no exposure or controls where no likely Gd deposits could be confirmed by our semi-quantitative elemental bioimaging approach. The time of symptom onset was very heterogeneous and not in all skin samples the IENFD was significantly reduced. However, all patients represented with length dependent neuropathic symptoms as the main diagnostic parameter of SFN.

Due to the high variability of Gd exposure in terms of dosage and time there was no correlation of dermal Gd deposits and exposure could be observed. A study analyzing skin biopsies short time after Gd exposure would be therefore interesting. Our data cannot provide the pathophysiological mechanism underlying the small fiber damage after Gd exposure. Possible explanations remain speculative and include mainly the unknown mechanisms of the Gd release from macrocyclic GBCA that are routinely used nowadays. However, our findings render the suggestion likely that GBCA might mediate neurotoxicity in susceptible patients. Further studies identifying factors that increase the risk of GBCA side effects on the peripheral nerve system would be of great benefit.

In iSFN patients with reported Gd exposure we were able to detect dermal Gd deposits. For this purpose, an elemental bioimaging approach was established using the LA-ICP-MSI method, which requires only minimal sample preparation and low amounts of tissue. For the application described here, a script-based semi-quantitative approach was developed utilizing the background Gd sensitivity as a normalization approach, and established NERs are expected to be comparable between different instruments and studies. Taken together, we describe a capable tool to detect traces of Gd in small tissue samples which may not solely used for the detection of Gd but may be transferred to the detection of other elements with low natural background in skin biopsies, like e. g. platinum (Pt) from anti-cancer drugs [31].

Surprisingly, we could not observe clear differences between macrocyclic and linear GBCAs with respect to the Gd deposition. In all animal studies [9, 10] there is clear evidence that (1) linear GBCAs release more Gd^3+^ ions into the tissue (higher deposition), and (2) due to the higher amount of free Gd ions the toxicity on small nerve fibers is more severe. However, the retrospective coding of the GBCA type in our study and three patients with mixed GBCA types in our iSFN patients sample the most prevalent type of GBCA exposure was only known for 15 patients (45%). In these patients, the IENFD was significantly reduced, however, the Gd deposition (NER classified as possible and likely) was not associated with this SFN pathology. Thus, to further investigate the proposed neurotoxic mechanism and any adverse effects on distal small nerve fibers in humans’ larger cohorts with detailed information about the GBCA exposures are needed.

Although the subgroup of iSFNne is rather small, no differences in QST parameters or pain characteristics could be detected between the iSFN groups. This is in accordance with previous studies showing that neuropathic pain derives from lesions of the somatosensory nervous system and cannot be linked to a specific underlying disease [32]. However, no correlations of QST and Gd deposits could be shown in our patients. However, further studies with larger number of patients are needed to further elucidate a possible connection.

### Limitations

Any attempt to perform a dose–response relationship using either the type of GBCA or the duration since the last GBCA exposure as a predictor for the Gd deposition in the skin biopsies or the neuropathological examinations was limited to a small number of patients providing the necessary information. On a purely descriptive level, no systematic association became obvious that could be interpreted as causal link between crucial characteristics of GBCA exposure (e.g. linear GBCAs release more Gd^3+^) and quantitative estimated of Gd deposition in tissue (NER). Furthermore, these crucial exposure characteristics could not be used to prove any causality of a Gd^3+^-related reduction of the IENFD in iSFN patients. Moreover, studies investigating the relation between the Gd skin deposits and the time and type of GBCA administration could further increase the application range of this method. Here, a better documentation of the GBCA exposures of the iSNF patients is needed.

## Conclusions

The previous findings of in vivo experiments with GBCA exposed mice [9] seems to be relevant for humans but further studies are needed to shed light on the mechanisms underlying this possible adverse side-effect of GBCAs. Nevertheless, our study showed that in iSFN patients with exposure to GBCAs dermal Gd deposits could be detected and IENFD was significantly reduced compared to iSFN patients without exposure to GBCAs and controls. However, the design of our study is not suitable to conclude that GBCA exposures are a risk factor for the development of iSFN. Here, a large prospective cohort study would be needed to clarify a causative role of Gd deposition in the etiology of iSFN.

## Supplementary Information

Below is the link to the electronic supplementary material.

Supp_Material_1: details about the elemental bioimaging using laser ablation-inductively coupled plasma-mass spectrometric imaging (LA-ICP-MSI) (PDF 593 KB)
