# Supplementary material for: Gadolinium contrast agents: dermal deposits and potential effects on epidermal small nerve fibers
Source: J Neurol. 2023 May 4;270(8):3981–91. doi: 10.1007/s00415-023-11740-z (PMC10344987; doi:10.1007/s00415-023-11740-z)
Supplement: Supplementary file 1 — Supp_Material_1: details about the elemental bioimaging using laser ablation-inductively coupled plasma-mass spectrometric imaging (LA-ICP-MSI) (PDF 593 KB) [file 415_2023_11740_MOESM1_ESM.pdf]

Semi-quantitative evaluation approach for gadolinium data

Due to the overall low Gd-concentrations, high number of samples, and low sample volume, commonly used analysis approaches to determine Gd concentrations were inapplicable. Instead, the density of Gd signals (meaning number of signals per unit of area) resulting from LA-ICP-TQMS analyses were utilized to assess the likelihood that a patient was administered a GBCA.

For the calculation of the signal density in the sample, a suitable input data set for the script needs to be generated and, thus, different sample shapes and sizes need to be accounted for (compare Supplemental Figure 1: Data selection). For this, all pixels of the considered Gd image are divided into two classes: sample pixels and background pixels. This is performed via the alpha map function of the software ImaJar (V3.64b, developed by Robin Schmid), which allows visualizing the distribution of one element depending on the intensity of another element. In this case, all Gd pixels, which have a spatially corresponding P intensity above a defined threshold, are defined as sample pixels. Consequently, pixels below the threshold are defined as background pixels. The P threshold is set as the lowest P intensity observed in the tissue. Additionally, cutting artifacts and tissue overlap are excluded manually. As a result, two lists, one containing all intensities of the sample pixels, the other containing all intensities of the background pixels, are generated for each sample and used as input data for the script.

In a next step, the data is cleaned from the impact of measurement parameters (compare Supplemental Figure 1: Signal transformation). Therefore, in the script, the data is transformed into a uniform format to allow for comparability between different analyses and studies. For the data transformation, each signal is divided by the intensity that is corresponding to the detection of a single count in the ICP-MS. For elements with a low background like Gd, this was defined as the smallest background intensity larger than zero.

To minimize the influence of single outliers, due to, e.g., dust particles on the tissue, the maximum intensity after the transformation is capped at a value of 4 and all higher values were set to 4. This means that the average intensity level is still accounted for in the data set, but individual outliers are no longer overrepresented and the focus remains on the signal density. It should be noted that this approach limits the scope of the script to samples with very low intensity levels but for other samples, conventional quantification is often a valid alternative.

The next step is deriving a comparable estimator from the transformed data sets that semi-quantitatively reflects the Gd concentration in the tissue (compare Supplemental Figure 1: Calculation). For this, the transformed signals are summed up individually for both lists. Afterwards, the influence of the number of pixels on the results is removed by normalizing the sums to the total number of pixels in the respective list. The resulting “event rate” already reflects the frequency and intensity of Gd signals in the map. However, it is still dependent on the sensitivity of the ICP-MS, which might vary between different instrument tunes and mass spectrometers. Hence, to compensate for those effects, the sample event rate is divided by the background event rate. The background is, thus, used to normalize each individual analysis run, which removes the influence of potential signal drifts between measurements. The resulting ratio was defined as the “Normalized Event Rate” (NER), which is a value that reflects the Gd signal density and intensity level and is mostly independent of tissue size and instrument parameters.

To derive a statement as to whether a patient has received prior GBCA administration or not. The NERs are classified in analogy to the 3σ- and 10σ-criteria (compare Supplemental Figure 1: Classification). If the NER of the sample (NER_{sample}) is lower than three times the NER of the control ($NER_{control}$), a previous GBCA

administration was defined to be "unlikely". If NER_{sample} is above 3 · $NER_{control}$ but is below 10 · $NER_{control}$, then it is "possible" that a GBCA was administered to the patient. Finally, if NER_{sample} is larger than 10 · $NER_{control}$, the patient is classified as "likely" to have received a GBCA administration. Because $NER_{control}$ is intended to reflect the natural background of Gd in tissue, a non-blinded analysis of a tissue sample from a healthy individual without prior GBCA administration was used for its determination.

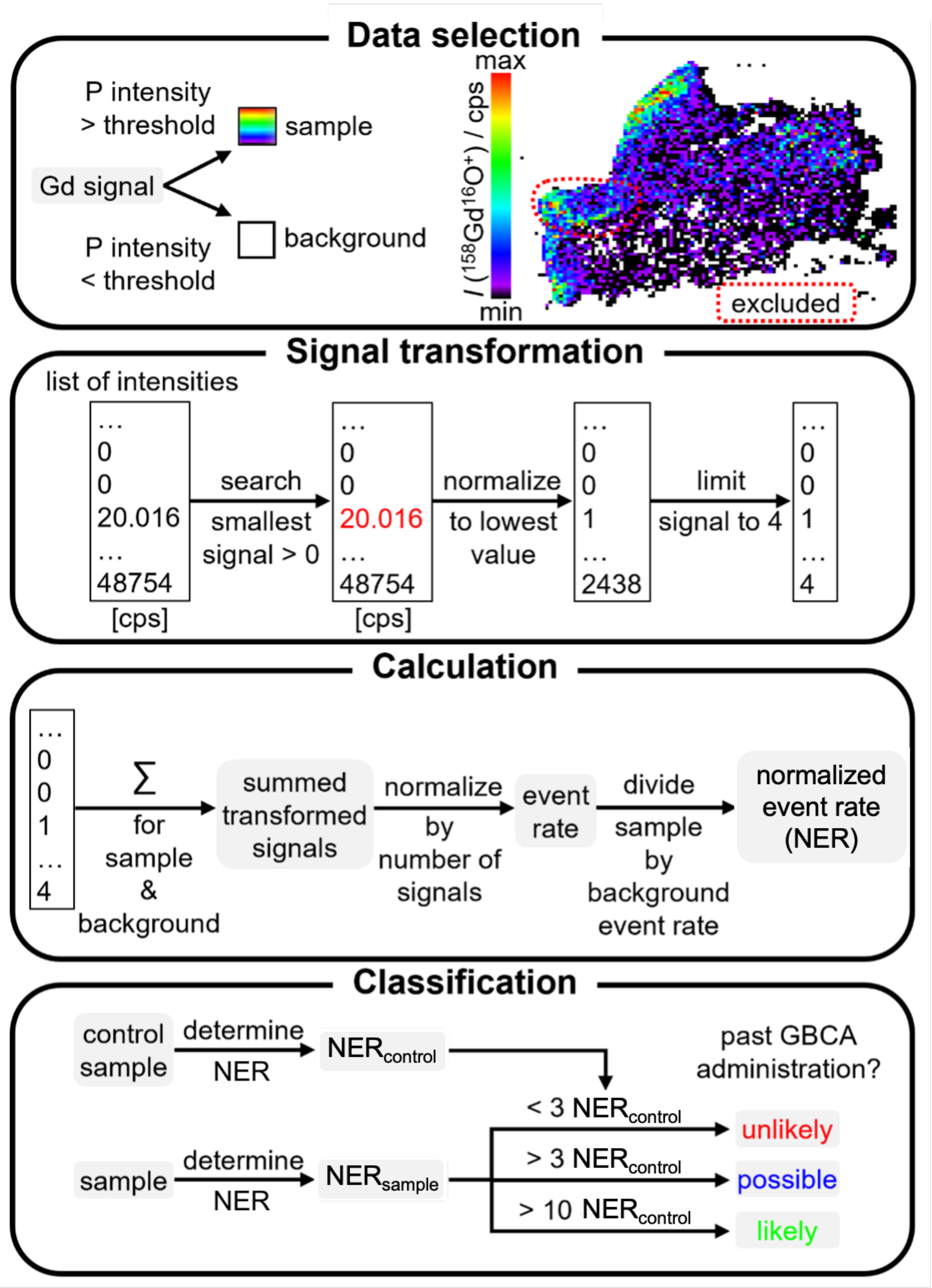

Supplemental Figure 1. Visualization of the utilized semi-quantitative evaluation approach to assess the likelihood of a GBCA administration via laser ablation-inductively coupled plasma-triple quadrupole mass spectrometry
